# Associations between Plasma Lipid Mediators and Chronic Daily Headache Outcomes in Patients Randomized to a Low Linoleic Acid Diet with or without Added Omega-3 Fatty Acids

**DOI:** 10.3390/metabo13060690

**Published:** 2023-05-25

**Authors:** Qing Shen, Jun Yang, Daisy Zamora, Mark Horowitz, Keturah R. Faurot, Beth A. MacIntosh, J. Douglas Mann, Bruce D. Hammock, Christopher E. Ramsden, Ameer Y. Taha

**Affiliations:** 1 Department of Food Science and Technology, College of Agriculture and Environmental Sciences, University of California, One Shields Avenue, Davis, CA 95616, USA; 2 Department of Entomology and Nematology & UCD Comprehensive Cancer Center, University of California, Davis, CA 95616, USA; 3 Lipid Peroxidation Unit, Laboratory of Clinical Investigation, National Institute on Aging, Baltimore, MD 21224, USA; 4 Department of Psychiatry, UNC School of Medicine, University of North Carolina at Chapel Hill, Chapel Hill, NC 27599, USA; 5 Department of Physical Medicine and Rehabilitation, Program on Integrative Medicine, University of North Carolina at Chapel Hill, Chapel Hill, NC 27599, USA; 6 Nutrition Research and Metabolism Core, North Carolina Translational Clinical Sciences Institute, University of North Carolina, Chapel Hill, NC 27599, USA; 7 Department of Neurology, University of North Carolina, Chapel Hill, NC 27599, USA; 8 West Coast Metabolomics Center, Genome Center, University of California, Davis, CA 95616, USA; 9 Laboratory of Membrane Biochemistry and Biophysics, National Institute on Alcohol Abuse and Alcoholism, National Institutes of Health, Bethesda, MD 20892, USA; 10 Center for Neuroscience, University of California, Davis, CA 95616, USA

**Keywords:** lipids, pain, psychological distress, quality of life, diet, fish

## Abstract

A previous report showed that 12-week lowering of dietary omega-6 linoleic acid (LA) coupled with increased omega-3 polyunsaturated fatty acid (PUFA) intake (H3-L6 diet) reduced headache frequency and improved quality of life in patients with chronic daily headaches (CDHs) compared to dietary LA reduction alone (L6 diet). The trial also showed that targeted dietary manipulation alters PUFA-derived lipid mediators and endocannabinoids. However, several additional classes of lipid mediators associated with pain in preclinical models were not measured. The current secondary analysis investigated whether the clinical benefits of the H3-L6 diet were related to changes in plasma unesterified PUFA-derived lipid mediators known to be involved in nociception, including prostanoids. Lipid mediators were measured by ultra-high-pressure liquid chromatography coupled with tandem mass-spectrometry. Compared to baseline, dietary LA lowering with or without added omega-3 fatty acids did not alter unesterified n-6 PUFA-derived lipid mediators, although several species derived from LA, di-homo-gamma-linolenic acid, and arachidonic acid were positively associated with headache frequency and intensity, as well as mental health burden. Alpha-linolenic acid (ALA)-derived metabolites were also associated with increased headache frequency and intensity, although they did not change from the baseline in either dietary group. Compared to baseline, docosahexaenoic acid (DHA)-derived epoxides were more elevated in the H3-L6 group compared to the L6 group. Diet-induced elevations in plasma DHA-epoxides were associated with reduced headache frequency, better physical and mental health, and improved quality of life (*p* < 0.05). Prostanoids were not detected, except for PGF2-alpha, which was not associated with any outcomes. This study demonstrates that diet-induced changes in DHA-epoxides were associated with pain reduction in patients with chronic headaches, whereas n-6 PUFA and ALA metabolites were associated with nociception. Lipid mediator associations with mental health and quality of life paralleled pain management outcomes in this population. The findings point to a network of multiple diet-modifiable lipid mediator targets for pain management in individuals with CDHs.

## 1. Introduction

Chronic daily headaches (CDHs), including both migraines and tension-type headaches, are characterized by frequent bouts of intense headaches that occur 15 or more days per month for at least 3 months [1,2]. CDHs affect ~3–5% of the population [1,2] and are associated with reduced quality of life and psychological distress due to high levels of anxiety, depression, and somatization [3,4]. Headache symptoms are treated with various classes of anti-inflammatory and anticonvulsant medications, which target nociception pathways (Reviewed in [5]). Although these drugs have varying degrees of clinical efficacy, they are non-curative. They are also ineffective in approximately 5% of patients who remain refractory to drug treatment [6], underscoring the need for new effective therapies.

Lipid autacoids (i.e., oxylipins) derived from lipoxygenase (LOX), cyclooxygenase (COX), cytochrome (CYP) P450, and soluble epoxide hydrolase (sEH) metabolism of polyunsaturated fatty acids (PUFAs) play an important role in pain regulation [7]. In rodents, linoleic acid (LA)-derived oxylipins promote nociception by activating the transient receptor potential vanilloid (TRPV)-1 pain receptor, while eicosapentaenoic acid (EPA, 20:5n-3) and docosahexaenoic acid (DHA, 22:6n-3)-derived oxylipins dampen nociception by blocking TRPV-1 activation [8,9,10,11,12]. CYP-derived epoxy oxylipins are generally anti- nociceptive, but they can further degrade into potentially more nociceptive diols by sEH [11]. For example, CYP-derived epoxides of arachidonic acid (AA) reduce nociception [13,14], whereas their sEH-derived diols are pro-nociceptive [15,16]. In humans, circulating concentrations of LA-derived oxylipins were observed to be higher in patients with Achilles tendinopathy compared to sex- and age-matched healthy controls [17] and were reported to be associated with increased pain scores in women with chronic neck pain [18].

Because PUFAs are precursors to bioactive lipid autacoids, targeted modifications of dietary PUFA composition have been used to manipulate both in vivo concentrations of LA, AA, EPA, and DHA [19], and their lipid-derived autacoids for CDH management [20,21]. In this regard, concomitant reductions in dietary LA (from 6.4% to 2.5% energy) and increases in EPA and DHA intake (from 47 to 1482 mg) were shown in a previous randomized, controlled pilot trial in a CDH population (the “CDH Trial”) to reduce plasma LA-derived oxylipins and to increase EPA- and DHA-derived oxylipins [21,22]. In the study, the 12-week low LA/high n-3 PUFA dietary intervention (H3-L6) reduced headache frequency and psychological distress and improved quality of life relative to baseline, compared to a low LA diet (L6) alone [20]. 

In this dietary intervention study, a number of unesterified oxylipins derived from the LOX and CYP-driven oxidation of LA, AA, alpha-linolenic acid (ALA, 18:3n-3), EPA, and DHA were altered by the L6 and/or H3-L6 diets, and several were found to be associated with headache outcomes [21,23]. Specifically, mono-hydroxylated metabolites of LA, ALA, and AA (i.e., oxidized metabolites containing one hydroxy group), as well as hydroxy-epoxy metabolites of LA, were found to be associated with increased headache frequency or intensity [23]. 

In contrast, hydroxylated metabolites of EPA, as well as hydroxylated (e.g., resolvins) and epoxidized metabolites of DHA, were associated with reduced headache frequency and intensity [23]. These observations were recently corroborated by another double-blind randomized trial, in which a similar H3-L6 diet increased circulating bound and free DHA-derived hydroxy metabolites (4,14 and 17-hydroxyDHA), and decreased headache intensity and frequency in patients with chronic migraine [24].

A limitation of the CDH Trial is that several key oxylipins potentially involved in nociception were not measured. These include AA- and gamma-linolenic acid (DGLA, 20:3n-6)-derived prostanoids, of which prostaglandin E2 (from AA) is a known promotor of headaches in humans [25] and PGE1 (from DGLA) has been implicated in pain relief in humans with peripheral vascular disease [26]. Additionally, epoxides of ALA, EPA, and AA, which have been shown to reduce pain in animals models [12,27] as well as pro- nociceptive fatty acid diols of sEH [15,16], have yet to be measured in relation to pain symptoms in humans. All of these lipid mediators have been shown to be affected by dietary LA lowering in rodent plasma and tissues [28,29], but it remains to be seen whether they change in humans on a low LA diet with or without added n-3 PUFAs and if such changes are associated with pain, psychological distress (i.e., anxiety and depression) secondary to perceived pain, and reduced quality of life [30]. 

The present secondary analysis sought to resolve these knowledge gaps by comprehensively quantifying PUFA-derived free oxylipins in archived plasma samples from the CDH Trial [31] and testing whether they relate to changes in headache frequency and intensity, psychological distress, and quality of life. In an exploratory manner, we also tested whether LA-derived ketones and trihydroxylated metabolites are related to headache outcomes because they were shown to change by LA-lowering in rat tissues [28,29] but have no known associations with pain outcomes. Our lipidomic measurements included oxylipins measured previously by other labs in the same cohort to confirm prior findings showing an association between DHA-derived free oxylipins and reduced headache frequency [23], while expanding our coverage to include more LA, DGLA, AA, ALA, EPA, and DHA-derived oxylipins thought to be involved in pain regulation or modifiable by dietary LA and EPA/DHA intake. Supplementary Table S1 identifies all compounds measured for the first time in this study. We hypothesized that dietary LA lowering coupled with increased n-3 PUFA intake will modify circulating prostanoids, PUFA-epoxides, and sEH-derived diols, and that these compounds will be associated with headache frequency and/or intensity, psychological distress, and quality of life. 

## 2. Patients and Methods

### 2.1. Patients and Dietary Methods

The CDH Trial was a randomized, 12-week trial conducted at the University of North Carolina at Chapel Hill (UNC) from April 2009 to November 2011. The trial tested the clinical and biochemical effects of a diet low in LA and high in n-3 PUFAs (i.e., the H3-L6 diet) compared to a low LA diet (i.e., the L6 diet) in a clinical population with refractory headaches [31]. The L6 intervention was designed to reduce dietary LA and AA and maintain an average US intake of n-3 PUFA, while the H3-L6 intervention was designed to concurrently reduce LA and increase n-3 PUFA (ALA, EPA and DHA) [32]. As previously reported, the L6 diet contained LA (≤2.5 en%), AA (60 mg), ALA (0.6 en%), and EPA + DHA (125 mg), while the H3-L6 diet contained LA (≤2.5 en%), AA(150 mg), ALA (>1.5 en%), and EPA + DHA (>1000 mg) [32]. A detailed description of the dietary interventions and clinical methods has been published [21,31]. The study protocol was approved by the UNC Institutional Review Board Subjects, and signed informed consent was obtained from each subject prior to participation. The trial is registered under ClinicalTrials.gov (NCT01157208).

Sixty-seven subjects with chronic headaches were randomized to either the L6 or H3-L6 diets for 12 weeks. The interventions were designed to provide equivalent: (1) amounts of study foods; (2) macronutrient and caloric intake; (3) interactions with the study investigators and dietitian; and (4) intensity and breadth of dietary advice and intervention materials [32]. A registered dietitian provided study foods meeting nutrient targets sufficient for two meals and two snacks per day, as well as dietary counseling both at randomization and at 2-week intervals. A study website included shopping guides, dining out guides, and recipes. To assess nutrient intakes, six unannounced telephone-administered 24 h recalls were administered for each participant—three during the baseline phase and three in the final four weeks of the intervention phase—as previously described [32].

A total of 56 of the 67 randomized participants completed the 12-week intervention phase, with 45 providing pre- and post-intervention plasma samples for free oxylipins analysis (23 in the L6 group and 22 in the H3-L6 group). Samples from the CDH Trial were collected between 8 December 2009 and 11 December 2011. The lipidomics analysis (described in Section 2.4) was performed in 2015. Baseline demographics and clinical characteristics were comparable in the two groups; 87% of randomized subjects were female. At baseline, participants averaged 23 headache days per month and 10 headache hours per day. An average of six different headache-related medications were being taken per subject.

### 2.2. Measures of Functional and Psychological Dimensions of Pain

The functional and psychological dimensions of pain were assessed by self-reported surveys at baseline and after 12 weeks of dietary intervention, as previously described [20]. In brief, the Headache Impact Test-6 (HIT-6) and the Physical Health Composite Score of the Medical Outcomes Study Short Forms 12 (SF-12) were used to evaluate the functional dimensions of pain, and the Mental Health Composite Score of SF-12 and the Brief Symptom Inventory (BSI-18) were used to evaluate the psychological dimensions of pain. A higher HIT-6 score indicates a higher level of headache-related disability. A higher SF-12 score indicates better quality of life. A higher BSI-18 score indicates greater psychological distress. 

### 2.3. Sample Collection

Fasting whole blood, drawn at baseline and again after 12 weeks of dietary intervention, was collected into ethylenediaminetetraacetic acid (EDTA) tubes. Samples were immediately centrifuged at 2960 rpm for 15 min at room temperature, and plasma aliquots were processed rapidly, placed on dry ice, and stored in a −80 °C freezer until analysis. Sample preparation and analyses were performed by investigators who were blinded to the study protocol and clinical data.

### 2.4. Free Oxylipin Extraction 

Free (unesterified) oxylipins were extracted by Waters Oasis HLB (3 cc, 60 mg sorbent; Waters, Milford, MA, USA) solid phase extraction (SPE) columns, as previously described [33,34,35]. Briefly, 10 µL of antioxidant solution, 10 µL of surrogate standard solution, and 1 mL of SPE buffer were added to 200 µL of plasma. The antioxidant solution contained 0.2 mg/mL of filtered butylated hydroxyl toluene (BHT), triphenyl phosphine (TPP), and EDTA in water/methanol (1:1 *v*/*v*). The surrogate standard stock solution contained 500 nM of d11-11(12)-EpETrE, d11-14,15-DiHETrE, d4-6-keto-PGF1α, d4-9-HODE, d4-LTB4, d4-PGE2, d4-TXB2, d6-20-HETE, and d8-5-HETE in LC-MS grade methanol. The SPE buffer contained 5% methanol and 0.1% acetic acid in ultrapure water.

The SPE columns were pre-washed, washed with one column volume of ethyl acetate, followed by two column volumes of methanol, and pre-conditioned with two column volumes of SPE buffer. The plasma samples were poured onto the columns and washed with two column volumes of SPE buffer. The columns were dried under vacuum suction (~20 psi) for 20 min. Free oxylipins were eluted from the column with 0.5 mL methanol and 1.5 mL ethyl acetate into a 2 mL centrifuge tube containing 6 µL of 30% glycerol in methanol. The oxylipin extract was dried in a speed-vac, reconstituted in 50 µL of LC-MS grade methanol containing 200 nM 1-cyclohexyl ureido, 3-dodecanoic acid as a recovery standard, and filtered by centrifugation using Ultrafree-MC-VV polyvinylidene fluoride filters (0.1 µm; Millipore, Bedford, MA, USA). 

### 2.5. Oxylipin Analysis by UPLC-MS/MS

As previously described [34,35,36], 86 oxylipin species were analyzed by ultra-high-pressure liquid chromatography-tandem mass spectrometry (UPLC-MS/MS) on an Agilent 1200SL (Agilent Corporation, Palo Alto, CA, USA) UPLC system coupled with a 4000 QTRAP tandem mass spectrometer (Applied Biosystems Instrument Corporation, Foster City, CA, USA). The system was equipped with an electrospray ionization source (Turbo V) and an Agilent Eclipse Plus C18 column (2.1 × 150 mm, 1.8 μm particle size; Agilent Technologies, Santa Clara, CA, USA). The instrument was operated in negative electrospray ionization mode and used optimized multiple reaction monitoring conditions to obtain parent and product ion pairs for each oxylipin.

The temperature of the auto-sampler and the column was kept at 4 °C and 50 °C, respectively. Ultrapure water containing 0.1% acetic acid was used as mobile phase A, while a acetonitrile/methanol/acetic acid (84/16/0.1 *v*/*v*) mixture was used as mobile phase B. Mobile phase B was held at 35% initially for 0.25 min and increased to 45% at 1 min, 55% at 3 min, 65% at 8.5 min, 72% at 12.5 min, 82% at 15 min, and 95% at 16.5 min, and held at 95% until 18 min. Mobile phase B was then decreased to 35% at 18.1 min and held at 35% until 21 min. The total run time was 21.5 min. The flow rate was maintained at 0.25 mL/min throughout the entire run.

Parent ions, product ions, and retention times (RTs) of the 34 oxylipins detected in this study are listed in Supplementary Table S2. Oxylipin concentrations were quantified by external standard curves after correcting for surrogate standard recoveries. The limit of quantification (LOQ) was defined as three times of the lowest observable concentration on the standard curve. Parameters of the method, including precision, accuracy, and extraction recovery have been previously described [34].

### 2.6. Data and Statistical Analysis

Statistical analyses were conducted using Stata version 13 (StataCorp LP, College Station, TX, USA) and followed an intention-to-treat principle. Oxylipin concentrations (nmol/L) are expressed as the median values with the interquartile ranges (IQR). Oxylipin concentration differences between baseline and after 12 weeks of dietary intervention within the H3-L6 group (n = 22) and within the L6 group (n = 23) were compared by Wilcoxon matched-pairs signed-rank tests. Oxylipin concentration differences after 12 weeks of dietary intervention between H3-L6 group (n = 22) and L6 group (n = 23) were compared by analysis of covariance (ANCOVA), adjusted for the respective baseline values. *p* values < 0.05 were considered significant. 

Correlations between oxylipin concentration changes and pain frequency and intensity were analyzed using Poisson regression and presented as % change in count for each standard deviation change in the respective oxylipin. Changes in oxylipin concentrations correlations with functional and psychological dimensions of pain were analyzed using linear regressions and presented as standard deviation change in Y (pain frequency and intensity and functional and psychological aspects of daily headaches) for each standard deviation change in X (plasma-free oxylipins).

## 3. Results

### 3.1. Effect of Dietary Intervention on Plasma-Free Oxylipins within H3-L6 and L6 Groups

A total of thirty-four out of eighty-six free oxylipins were detected in plasma, including six derived from ALA, seven derived from DHA, nine from LA, one from DGLA, and eleven from AA. EPA-derived oxylipins were measured but not detected because of low concentrations (below LOQ), which is consistent with a previous study in humans showing low detectability of EPA-derived metabolites [37]. PGF2a was the only detected prostanoid. Supplementary Table S1 shows oxylipins analyzed in our current and prior dietary intervention studies. There were 40 new compounds measured in this study compared to the prior study in the same cohort [21,23,24,38]. 

Table 1 shows plasma-free oxylipin concentrations derived from n-3 and n-6 PUFAs at baseline and after 12 weeks of dietary intervention. Compared to baseline, the L6 intervention (n = 23) significantly increased plasma DHA-derived 19,20-DiHDPE, AA-derived PGF2a, and LA-derived 9-oxo-ODE by 48%, 22%, and 29%, respectively (*p* < 0.05). Compared to baseline, the H3-L6 intervention (n = 22) significantly increased DHA-derived 13(14)-EpDPE, 16(17)-EpDPE, 19(20)-EpDPE, 10,11-DiHDPE, 16,17-DiHDPE, and 19,20-DiHDPE by 79~207% (*p* < 0.05), and decreased AA-derived 8,9-DiHETrE and LA-derived 9,10,13-TriHOME by 38% and 20%, respectively (*p* < 0.05). Similar to the L6 diet, the H3-L6 diet also increased PGF2a concentration by 21% compared to baseline, but this increase was borderline significant (*p* = 0.05).

Both interventions combined, produced marked changes in plasma n-3 and n-6 PUFA-derived oxylipins as shown in Table 1. Compared to baseline, the combined interventions (n = 45) significantly increased plasma-free DHA-derived 13(14)-EpDPE, 16(17)-EpDPE, 19(20)-EpDPE, 10,11-DiHDPE, 13,14-DiHDPE, 16,17-DiHDPE, and 19,20-DiHDPE by 37~80% (*p* < 0.05), as well as ALA-derived 15,16-DiHODE and AA-derived PGF2a by 31% and 22%, respectively (*p* < 0.05). 

### 3.2. Effect of Dietary Intervention on Plasma-Free Oxylipins between the H3-L6 and L6 Groups

There were no significant differences in plasma-free oxylipins between the H3-L6 and L6 groups at baseline (*p* > 0.05). After 12 weeks of dietary intervention (Table 1), the H3-L6 intervention resulted in significantly greater increases in DHA-derived 16(17)-EpDPE and 19(20)-EpDPE, and significantly more reductions in AA-derived 8(9)-EpETrE and LA-derived 9-oxo-ODE compared to the L6 intervention (*p* < 0.05).

### 3.3. Association between 12-Week Changes in Plasma-Free Oxylipins and Pain Frequency and Intensity

Associations between 12-week changes in plasma-free oxylipins and pain frequency (headache days per month) and intensity (headache hours per day) are shown in the first two columns of Table 2 (n = 45). For each standard deviation increase in plasma DHA-derived 16(17)-EpDPE, an 18% reduction in the number of headache days per month (*p* < 0.0001) and a 20% reduction in the number of headache hours per day (*p* = 0.0036) was observed. For each standard deviation increase in plasma DHA-derived 19(20)-EpDPE, a 12% reduction in the number of headache days per month (*p* = 0.0032) was observed. Additionally, each standard deviation increases in ALA-derived 9(10)-EpODE and 9-HOTrE was associated with a 12% (*p* = 0.0384) and 18% (*p* < 0.0001) increase in the number of headache hours per day and headache days per month, respectively. 

With regard to n-6 PUFAs, for each standard deviation increase in plasma AA-derived 8-HETE, a 12% significant reduction in the number of headache days per month (*p* = 0.0102) and a 17% significant reduction in the number of headache hours per day (*p* = 0.0305) was observed. However, each standard deviation increase in plasma AA-derived 11-HETE, 12-HETE, and 15-HETE was associated, respectively, with a significant 14%, 15%, and 11% increase in the number of headache days per month (*p* < 0.05). Each standard deviation increase in plasma-free n-6 DGLA-derived 15(S)-HETrE was associated with an 8.7% increase in the number of headache days per month (*p* = 0.0308). Similar associations were observed for LA-derived metabolites, where increases in 9-HODE, 13-HODE, 9-oxo-ODE, 9,10,13-TriHOME, and 9,12,13-TriHOME were associated with a 28%, 21%, 18%, 6.3%, and 6.1% increase in the number of headache days per month, respectively (*p* < 0.05). 

### 3.4. Association between 12-Week Changes in Plasma-Free Oxylipins and Functional Dimensions of Pain

As shown in Table 2 (third and fourth columns), increases in circulating DHA-derived 16(17)-EpDPE and 19(20)-EpDPE were related to lower HIT-6 scores (*p* < 0.05) and higher SF-12 (physical) scores (*p* < 0.05), reflecting improved quality of life. Similarly, each standard deviation increase in AA-derived 8,9-DiHETrE was correlated with lower HIT-6 scores, reflecting better quality of life (*p* = 0.033). In contrast, each standard deviation increase in LA-derived 9-oxo-ODE was related to lower SF-12 physical scores (i.e., more headache impact on quality of life; *p* = 0.0381).

### 3.5. Association between 12-Week Changes in Plasma-Free Oxylipins and Psychological Dimensions of Pain

As shown in Table 2, each standard deviation increase in LA-derived 9,10,13-TriHOME, a mediator highly associated with more headache days per month, was related to lower SF-12 mental scores (i.e., worse mental health; *p* = 0.0296). No other significant associations were observed between changes in free oxylipins and mental health or brief symptom inventory (markers of psychological distress).

### 3.6. Summary of Findings

Compared to the L6 diet, the H3-L6 diet increased free DHA-derived 16(17)-EpDPE and 19(20)-EpDPE and reduced AA-derived 8(9)-EpETrE and LA-derived 9-oxo-ODE in plasma. 

Diet-induced increases in DHA-derived epoxide concentrations (i.e., 16(17)-EpDPE and 19(20)-EpDPE) were associated with significant reductions in monthly and daily headache frequency and improved physical dimensions of pain (higher SF-12 physical scores) and quality of life (lower HIT-6 scores), consistent with prior findings in the same cohort [23]. ALA-derived 9(10)-EpODE and 9-HOTrE, LA-derived 9-HODE, 13-HODE, 9-oxo-ODE, 9,10,13-TriHOME and 9,12,13-TriHOME, DGLA-derived-15(S)-HETrE, and AA-derived 11-, 12-, and 15-HETE were associated with increased headache frequency or intensity. 8-HETE was associated with reduced headache frequency and intensity. The 9-HOTrE and 9-HODE associations are consistent with previous findings [23], whereas the 11-HETE associations are opposite to what has previously been reported [23]. Associations between ALA-derived 9(10)-EpODE, LA-derived 9-oxo-ODE, 9,10,13-TriHOME and 9,12,13-TriHOME, and DGLA-derived-15(S)-HETrE and headache outcomes are new. 

Additionally, 9-oxo-ODE was associated with reduced quality of life and 8,9-DiHETrE with better quality of life. 9,10,13-TriHOME was associated with worse mental health. All of these are new associations linking oxylipins to quality of life or psychological distress (mental health). 

## 4. Discussion

In the present study, we identified new associations between circulating ALA, LA, DGLA, and AA-derived oxylipins and headache outcomes in a cohort of CDH patients randomized to a low LA diet with or without n-3 PUFAs for 12 weeks. Specifically, ALA-derived 9(10)-EpODE, LA-derived 9-oxo-ODE, 9,10,13-TriHOME and 9,12,13-TriHOME, DGLA-derived-15(S)-HETrE, and AA-derived 11-, 12-, and 15-HETE were positively associated with headache frequency or intensity. LA-derived 9-oxo-ODE and 9,10,13-TriHOME were associated with reduced quality of life and worse mental health, respectively, whereas AA-derived 8,9-DiHETrE, a product of sEH-mediated degradation of 8(9)-EpETrE, was associated with better quality of life. Additionally, we confirmed previously reported associations between circulating ALA-derived 9-HOTrE and LA-derived 9-HODE and greater headache frequency, as well as between DHA-derived epoxides (16(17)-EpDPE and 19(20)-EpDPE) and lower headache frequency and improved functional dimensions of pain and quality of life [23]. Plasma PGE2, other prostanoids (except for PGF2-alpha), and EPA-derived metabolites were not detected in this study.

Except for DHA-derived metabolites, many of the lipid mediators that were associated with headache frequency/pain, psychological distress, and quality of life were minimally altered by the L6 and H3-L6 diets. Thus, compared to baseline, the combined L6 and H3-L6 interventions (n = 45) significantly increased plasma-free DHA-derived 13(14)-EpDPE, 16(17)-EpDPE, 19(20)-EpDPE, 10,11-DiHDPE, 13,14-DiHDPE, 16,17-DiHDPE, and 19,20-DiHDPE by 37~80%, without altering free LA-derived oxylipins. The lack of changes in LA metabolites is surprising given that LA intake, the precursor to LA-derived oxylipins, was lowered from 7.4% and 6.4% to 2.4% and 2.5% energy in the L6 and H3-L6 dietary groups, respectively [21]. In a prior analysis performed in the same cohort, dietary LA-lowering for 12 weeks reduced circulating LA-derived metabolites by ~20% [21,22]; however, measurements were performed on the ‘total’ LA-metabolite pool consisting of both free and esterified oxylipins. In this study, we only measured free oxylipins—the bioactive pool that mediates nociception [39]. Free oxylipins are quantitatively minor (<10%) in plasma compared to esterified oxylipins [40,41] because of their shorter half-life [42]. Our findings suggest that the free oxylipin pool may be less responsive to dietary LA changes compared to esterified oxylipins. 

The L6 diet alone resulted in minimal changes in circulating free oxylipins after the 12-week intervention period compared to baseline; it only increased DHA-derived 19,20-DiHDPE, AA-derived PGF2a, and LA-derived 9-oxo-ODE. This observation differs from rodent studies where significant reductions in free LA-derived metabolites and increases in EPA- and DHA-derived epoxides and diols were seen in plasma after 8 to 15 weeks of dietary LA lowering [28,43]. Thus, the response of rodents to a low LA diet appears to occur within a short period of time (weeks) compared to humans, where it may take many months to achieve a response due to species differences in the turnover of LA-derived oxylipins from unesterified LA. Indeed, in rats, 15 weeks of LA-lowering significantly reduced both esterified and unesterified LA and its oxylipin metabolites [28,44], whereas in humans, LA-lowering for 12 weeks reduced esterified LA without altering unesterified LA—the direct precursor to LA-derived oxylipins [19]. Prolonged dietary LA lowering in humans may be necessary to reduce circulating unesterified LA and its oxylipin metabolites.

The H3-L6 diet had more profound effects on plasma oxylipins compared to the L6 diet, and also relative to published reports on the effects of fish oil intake on plasma oxylipins. In this regard, the H3-L6 diet increased DHA-derived epoxides (13(14)-EpDPE, 16(17)-EpDPE, and 19(20)-EpDPE) and diols (10,11-DiHDPE, 16,17-DiHDPE and 19,20-DiHDPE) and lowered n-6 PUFA derived metabolites of AA (8,9-DiHETrE) and LA (9,10,13-TriHOME) compared to baseline. In humans, fish oil supplementation for 12 weeks did not alter free DHA-derived epoxides and diols in plasma [41], consistent with the suggestion that LA lowering coupled with increased EPA/DHA intake is more effective in increasing DHA turnover into their pro-resolving epoxide metabolites compared to EPA/DHA intake alone [45]. Notably, the observed reductions in n-6 PUFA-derived oxylipins (9,10,13-TriHOME and 8,9-DiHETrE) in the H3-L6 diet were not seen in the L6 diet. This implies an interaction between dietary LA lowering and increased n-3 PUFA intake, where the combined approach appears to be more effective in reducing n-6 PUFA-derived oxylipins compared to LA lowering alone (Reviewed in [46]). Future studies are needed to explore the mechanisms underlying this novel effect of H3-L6 intake.

In the present study, we confirmed previously reported associations between 9-HODE and 9-HOTrE and increased headache frequency, as well as between DHA-epoxides and reduced headache frequency in the same cohort [23]. Notably, different extraction protocols and LC-MS/MS systems were used in the present study compared to the prior study. Thus, the fact that our primary observations were reproduced here strengthens the evidence linking DHA-epoxides, LA-derived 9-HODE, and ALA-derived 9-HOTrE to headache outcomes, and indicates that different analytical approaches may be used (e.g., different mass-spec systems) to derive the same conclusion. 

The inverse association between DHA-epoxides and headache frequency adds to the body of pre-clinical evidence showing that free DHA-epoxides block pain in rodents [27]. It also corroborates prior observations showing that plasma hydroxylated metabolites of free EPA (18-hydroxyeicosapentaenoic acid/18-HEPE) and DHA (17-hydroxydocosahexaenoic acid/17-HDHA) are associated with pain reduction [21]. This suggests that both hydroxylated and epoxidized derivatives of EPA and DHA are anti-nociceptive in humans.

Several novel associations were found between oxylipins and headache frequencies in this study. ALA-derived 9(10)-EpODE, LA-derived 13-HODE, 9-oxo-ODE, 9,10,13-TriHOME and 9,12,13-TriHOME, DGLA-derived-15(S)-HETrE, and all AA-derived 11-, 12-, and 15-HETEs were associated with increased headache frequency. The observed link between LA-derived HODEs and AA-derived HETEs and headache frequency is in agreement with their established role in pain sensitization [17,18,47]. To our knowledge, this is the first study to show the involvement of ALA-derived 9(10)-EpODE, LA-derived 9-oxo-ODE, 9,10,13-TriHOME and 9,12,13-TriHOME, and DGLA-derived-15(S)-HETrE in pain modulation. Future studies should explore the mechanisms by which these lipid mediators modulate pain thresholds.

Changes in several lipid mediators were associated with physical and mental health and quality of life. Specifically, reductions in LA-derived 9-oxo-ODE and LA-derived 9,10,13 TriHOME were related to better physical scores and mental health scores, respectively (Table 2). Increases in circulating DHA-derived 16(17)-EpDPE and 19(20)-EpDPE were also related to better physical scores and improved quality of life. Because these lipid mediators were associated with reduced headache frequency and/or intensity, improvements in physical and mental health domains and quality of life may be secondary to better pain management in this population. 

Similarly, AA-derived 8,9-DiHETrE, a product of the sEH-mediated degradation of 8(9)-EpETrE, was associated with improvements in the HIT-6 scale. This is somewhat consistent with the inverse association between 8,9-DiHETrE and headache frequency, which approached statistical significance (*p* = 0.0671). The HIT-6 measures how headaches interfere with daily activities. Our data implicate 8,9-DiHETrE in reduced disability secondary to headaches. 

The limitations of this study include the relatively small size and the lack of a control group that was maintained on population-level LA and n-3 PUFA intakes of 7% and <1% energy, respectively. The strengths include the comprehensive coverage of unesterified lipid autacoids, as well as the within-subject design, which allowed us to capture diet-induced changes in free oxylipins after 12 weeks of intervention. 

In conclusion, this study showed that 12-week dietary LA lowering with or without increased n-3 PUFA intake resulted in increased free DHA-epoxides in the plasma of patients with CDHs. These changes were associated with better headache control and functional improvements in physical and mental health, as well as reduced headache interferences in daily activities. Novel associations between LA, ALA, DGLA, and AA-derived oxylipins and headache outcomes were also discovered, although plasma concentrations of many of these mediators were not significantly impacted by the L6 and H3-L6 diets. Amongst these mediators, 9-oxo-ODE was associated with reduced quality of life, 8,9-DiHETrE with better quality of life, and 9,10,13-TriHOME with worse mental health, indicating that some markers of poor headache control translated to elevated psychological distress and reduced quality of life. Our findings point to multiple bioactive lipid autacoid networks that predict headache frequency in CDH patients and could potentially serve as new biomarkers or targets for better pain management. 

## Figures and Tables

**Table 1:** Plasma-free oxylipins derived from n-3 and n-6 fatty acids at baseline and after 12 weeks of dietary intervention.

Compound	H3-L6 Group (n = 22) ^1^			L6 Group (n = 23) ^1^			Both Groups (n = 45)		Between-Group (Week 12) ^5^	
nmol/L	Baseline ^2^ Median (IQR)	Median Percent Change ^3^	Pre-vs-Post *p*-Value ^4^	Baseline ^2^ Median (IQR)	Median Percent Change ^3^	Pre-vs-Post *p*-Value ^4^	Median Percent Change ^3^	Pre-vs-Post *p*-Value ^4^	Coef	*p*-Value
**Docosahexaenoic acid (DHA, 22:6 n-3)-derived oxylipins**										
13(14)-EpDPE	**0.01 (0.00, 0.01)**	**206.73**	**0.006**	0.01 (0.01, 0.03)	2.83	0.465	**61.09**	**0.009**	0.01	0.257
16(17)-EpDPE	**0.54 (0.22, 1.00)**	**165.00**	**0.002**	0.89 (0.33, 1.38)	9.45	0.715	**80.34**	**0.005**	**0.77**	**0.023**
19(20)-EpDPE	**1.22 (0.85, 2.03)**	**183.91**	**0.002**	1.54 (0.81, 2.33)	29.76	0.362	**72.24**	**0.001**	**1.44**	**0.027**
10,11-DiHDPE	**0.15 (0.08, 0.22)**	**178.22**	**0.024**	0.15 (0.07, 0.26)	13.93	0.484	**46.26**	**0.020**	−0.25	0.894
13,14-DiHDPE	0.19 (0.17, 0.26)	66.18	0.067	0.17 (0.13, 0.26)	32.87	0.059	**47.56**	**0.009**	−1.46	0.473
16,17-DiHDPE	**0.43 (0.19, 0.54)**	**79.30**	**0.042**	0.46 (0.27, 0.59)	−6.17	0.523	**36.60**	**0.023**	−1.49	0.672
19,20-DiHDPE	**2.82 (2.27, 3.97)**	**79.70**	**0.031**	**2.56 (1.84, 3.28)**	**48.38**	**0.036**	**48.39**	**0.003**	−28.16	0.257
**α-linolenic acid (ALA, 18:3 n-3)-derived oxylipins**										
9-HOTrE	0.71 (0.38, 1.08)	13.78	0.685	0.48 (0.39, 0.66)	35.11	0.153	14.97	0.531	0.03	0.850
9(10)-EpODE	2.14 (0.90, 3.23)	17.82	0.223	2.70 (0.90, 6.99)	72.95	0.563	22.56	0.229	−1.29	0.411
12(13)-EpODE	1.20 (0.56, 1.86)	−13.06	0.615	1.79 (0.88, 3.49)	2.55	0.543	1.11	0.538	−0.60	0.413
15(16)-EpODE	7.54 (4.30, 11.96)	28.56	0.338	7.95 (4.57, 16.49)	42.46	0.248	31.24	0.144	−1.25	0.652
9,10-DiHODE	0.20 (0.13, 0.70)	13.16	0.733	0.25 (0.15, 0.38)	7.25	0.162	7.25	0.439	−1.69	0.308
15,16-DiHODE	12.59 (7.01, 22.68)	34.51	0.072	12.40 (9.25, 17.63)	26.69	0.144	**30.75**	**0.032**	−79.37	0.356
**Arachidonic acid (AA, 20:4 n-6)-derived oxylipins**										
5-HETE	0.78 (0.55, 1.32)	−19.10	0.168	1.01 (0.72, 1.31)	−7.00	0.346	−13.63	0.098	−0.05	0.753
8-HETE	0.34 (0.14, 0.73)	−16.10	0.408	0.41 (0.26, 1.14)	−46.62	0.191	−20.59	0.174	−0.02	0.835
11-HETE	0.27 (0.15, 0.49)	−2.98	0.935	0.41 (0.17, 0.54)	−13.64	0.212	−13.64	0.433	0.004	0.961
12-HETE	2.43 (1.51, 3.67)	−17.48	0.445	2.44 (1.58, 4.27)	17.14	0.670	3.32	0.400	−0.26	0.611
15-HETE	1.33 (0.83, 1.74)	3.88	0.808	1.44 (0.75, 2.52)	−9.74	0.503	−7.08	0.765	−0.04	0.896
8,9-DiHETrE	**0.43 (0.34, 0.56)**	**−37.52**	**0.026**	0.39 (0.29, 0.61)	2.02	0.648	−23.55	0.234	−4.12	0.259
11,12-DiHETrE	0.72 (0.56, 0.96)	−17.73	0.115	0.64 (0.53, 0.80)	7.10	0.201	−4.28	0.826	−6.37	0.152
8(9)-EpETrE	0.81 (0.36, 1.26)	−17.17	0.592	0.99 (0.48, 1.74)	35.23	0.429	4.97	0.861	**−0.61**	**0.035**
11(12)-EpETrE	1.82 (1.26, 2.89)	13.01	0.884	3.15 (1.17, 6.21)	21.00	0.670	19.37	0.648	−1.06	0.209
14(15)-EpETrE	1.74 (1.11, 2.79)	−22.84	0.961	2.98 (1.04, 4.65)	29.91	0.523	8.00	0.731	−0.77	0.226
PGF2a	**6.59 (5.16, 8.62)**	**21.26**	**0.050**	**5.90 (4.23, 6.65)**	**22.06**	**0.048**	**22.06**	**0.006**	−0.57	0.785
**Di-homo-gamma-linolenic acid (DGLA, 20:3 n-6)-derived oxylipins**										
15(S)-HETrE	1.41 (0.97, 1.72)	−25.76	0.236	1.30 (0.97, 1.67)	−8.68	0.394	−9.26	0.160	−0.09	0.662
**Linoleic acid (LA, 18:2 n-6)-derived oxylipins**										
9-HODE	6.68 (4.82, 8.89)	−10.55	0.338	5.96 (4.53, 7.78)	23.26	0.144	6.99	0.835	−0.85	0.460
13-HODE	13.53 (9.62, 19.78)	−17.60	0.115	11.51 (9.49, 15.79)	12.25	0.378	−0.14	0.531	−1.93	0.448
9-oxo-ODE	7.15 (2.84, 10.37)	−27.91	0.140	**4.93 (3.12, 8.66)**	**28.89**	**0.042**	2.63	0.897	**−2.67**	**0.033**
9(10)-EpOME	82.58 (41.10, 128.79)	−2.31	0.884	112.74 (53.98, 256.28)	7.13	0.927	1.75	0.915	−77.80	0.121
12(13)-EpOME	22.52 (14.62, 32.13)	−6.08	0.527	29.34 (13.69, 58.24)	1.19	0.976	−1.06	0.680	−14.83	0.166
9,10-DiHOME	3.73 (2.70, 5.33)	−18.19	0.372	3.55 (2.53, 7.85)	24.12	0.362	−6.42	0.977	−39.49	0.243
12,13-DiHOME	7.89 (5.68, 15.66)	−18.21	0.445	7.37 (4.84, 14.01)	−0.18	0.761	−8.12	0.748	−56.20	0.352
9,10,13-TriHOME	**3.03 (2.50, 6.02)**	**−19.57**	**0.014**	3.38 (2.39, 5.96)	−3.93	0.976	−11.36	0.105	−11.40	0.362
9,12,13-TriHOME	5.04 (4.46, 10.97)	3.66	0.987	6.38 (4.07, 11.10)	−12.67	0.563	−4.69	0.460	−20.35	0.347

^1^ H3-L6 Group, high n-3 plus low n-6 diet, n = 22; L6 Group, low n-6 diet, n = 23. There were no differences between diet groups at baseline (*p* > 0.05). ^2^ Oxylipin concentrations (nmol/L) at baseline are expressed as the median values with the interquartile ranges (IQR). ^3^ Median of the percent change from baseline to week 12 calculated for each subject. ^4^ Wilcoxon matched-pairs signed-rank tests were used for intragroup comparisons. ^5^ Effect of dietary intervention on plasma-free oxylipins at 12 weeks were compared by analysis of covariance, adjusting for baseline. Coef, coefficients. Data in bold represent *p* values less than 0.05.

**Table 2:** Association between 12-week changes in plasma-free oxylipins and pain frequency and intensity, functional aspects of chronic daily headache (CDH), and psychological aspects of CDH ^1^.

	Pain Frequency and Intensity ^2^				Functional Dimensions of Pain ^3^				Psychological Dimensions of Pain ^3^			
Oxylipins	Headache Days/Month		Headache Hours/Day		HIT-6 ^4^		SF-12 (Physical) ^5^		SF-12 (Mental) ^5^		BSI-18 ^6^	
	Coef	*p*-Value	Coef	*p*-Value	Coef	*p*-Value	Coef	*p*-Value	Coef	*p*-Value	Coef	*p*-Value
**Docosahexaenoic acid (DHA, 22:6 n-3)-derived oxylipins**												
13(14)-EpDPE	1.8%	(0.6483)	2.0%	(0.7458)	−0.23	(0.0837)	0.20	(0.1302)	0.11	(0.4086)	−0.19	(0.0965)
16(17)-EpDPE	**−18%**	**(<0.0001)**	**−20%**	**(0.0036)**	**−0.37**	**(0.0029)**	**0.33**	**(0.0088)**	0.21	(0.1103)	−0.20	(0.0874)
19(20)-EpDPE	**−12%**	**(0.0032)**	−9.4%	(0.1530)	**−0.33**	**(0.0134)**	**0.31**	**(0.0171)**	0.12	(0.3895)	−0.21	(0.0966)
10,11-DiHDPE	4.4%	(0.1971)	6.0%	(0.2679)	0.15	(0.2558)	−0.23	(0.0835)	−0.11	(0.4099)	0.05	(0.7115)
13,14-DiHDPE	2.3%	(0.5229)	2.0%	(0.7236)	0.16	(0.2338)	−0.21	(0.0948)	−0.12	(0.3610)	0.10	(0.4271)
16,17-DiHDPE	5.5%	(0.1078)	7.2%	(0.1771)	0.16	(0.2406)	−0.07	(0.5902)	−0.17	(0.2223)	0.09	(0.4899)
19,20-DiHDPE	4.1%	(0.2537)	5.7%	(0.2930)	0.07	(0.5764)	−0.05	(0.7244)	−0.19	(0.1500)	0.08	(0.5385)
**α-linolenic acid (ALA, 18:3 n-3)-derived oxylipins**												
9-HOTrE	**18%**	**(<0.0001)**	0.57%	(0.9105)	0.08	(0.5477)	−0.19	(0.1159)	0.11	(0.4280)	−0.05	(0.7179)
9(10)-EpODE	7.3%	(0.0511)	**12%**	**(0.0384)**	0.15	(0.2624)	−0.05	(0.7379)	0.04	(0.8008)	0.05	(0.7033)
12(13)-EpODE	4.0%	(0.2940)	6.7%	(0.2528)	0.09	(0.5221)	0.01	(0.9594)	0.03	(0.8493)	0.05	(0.7188)
15(16)-EpODE	4.7%	(0.2214)	7.5%	(0.1993)	0.08	(0.5717)	0.01	(0.9670)	0.10	(0.4889)	<0.01	(0.9861)
9,10-DiHODE	0.033%	(0.9930)	−2.8%	(0.6255)	0.07	(0.6035)	−0.06	(0.6533)	0.06	(0.6612)	0.07	(0.5826)
15,16-DiHODE	0.54%	(0.8795)	1.2%	(0.8312)	0.12	(0.3581)	−0.04	(0.7920)	−0.10	(0.4819)	0.11	(0.3997)
**Arachidonic acid (AA, 20:4 n-6)-derived oxylipins**												
5-HETE	1.6%	(0.7222)	−1.6%	(0.8172)	0.01	(0.9426)	−0.12	(0.3785)	0.25	(0.0928)	−0.08	(0.5711)
8-HETE	**−12%**	**(0.0102)**	**−17%**	**(0.0305)**	−0.11	(0.4388)	−0.10	(0.4683)	0.16	(0.2527)	0.07	(0.6100)
11-HETE	**14%**	**(0.0006)**	6.1%	(0.2755)	0.07	(0.6195)	−0.18	(0.2082)	<0.01	(0.9824)	0.18	(0.1888)
12-HETE	**15%**	**(0.0001)**	9.4%	(0.0893)	0.15	(0.2525)	−0.11	(0.4289)	−0.06	(0.6640)	0.13	(0.3103)
15-HETE	**11%**	**(0.0090)**	−2.8%	(0.6514)	−0.13	(0.3239)	−0.09	(0.5070)	0.08	(0.5821)	0.07	(0.6061)
8,9-DiHETrE	−8.4%	(0.0671)	−9.1%	(0.2630)	**−0.28**	**(0.0330)**	−0.03	(0.8149)	0.02	(0.8790)	0.05	(0.6827)
11,12-DiHETrE	−2.4%	(0.5243)	−2.6%	(0.6650)	0.09	(0.4836)	−0.02	(0.9063)	0.05	(0.7491)	−0.04	(0.7481)
8(9)-EpETrE	2.1%	(0.6445)	9.5%	(0.1691)	0.09	(0.5347)	0.06	(0.6779)	−0.09	(0.5303)	0.03	(0.8261)
11(12)-EpETrE	−0.81%	(0.8467)	5.2%	(0.4154)	0.06	(0.6491)	0.08	(0.5437)	−0.01	(0.9487)	<0.01	(0.9961)
14(15)-EpETrE	−1.7%	(0.6988)	12%	(0.0652)	0.03	(0.8374)	0.03	(0.8291)	<0.01	(0.9961)	−0.01	(0.9561)
PGF2α	0.029%	(0.9934)	2.7%	(0.5889)	−0.06	(0.6586)	0.02	(0.8749)	0.01	(0.9673)	−0.09	(0.4417)
**Di-homo-gamma-** **linolenic acid (DGLA, 20:3 n-6)-derived oxylipins**												
15(S)-HETrE	**8.7%**	**(0.0308)**	6.6%	(0.2767)	0.13	(0.3464)	−0.24	(0.0621)	0.15	(0.2840)	0.07	(0.5864)
**Linoleic acid (LA, 18:2 n-6)-derived oxylipins**												
9-HODE	**28%**	**(<0.0001)**	3.0%	(0.5760)	0.14	(0.2949)	−0.18	(0.1752)	0.17	(0.2151)	−0.11	(0.4081)
13-HODE	**21%**	**(<0.0001)**	3.6%	(0.4991)	0.11	(0.4104)	−0.20	(0.1229)	0.05	(0.7338)	−0.07	(0.5639)
9-oxo-ODE	**18%**	**(0.0002)**	4.5%	(0.5133)	0.18	(0.1809)	**−0.29**	**(0.0381)**	−0.01	(0.9367)	0.06	(0.6381)
9(10)-EpOME	3.6%	(0.3374)	6.2%	(0.2735)	0.08	(0.5686)	0.07	(0.6058)	−0.06	(0.6874)	0.08	(0.5263)
12(13)-EpOME	1.3%	(0.7251)	7.1%	(0.2143)	0.06	(0.6699)	<0.01	(0.9946)	−0.01	(0.9720)	0.05	(0.6912)
9,10-DiHOME	−3.5%	(0.3647)	−6.9%	(0.2766)	0.06	(0.6422)	0.04	(0.7489)	0.03	(0.8580)	0.09	(0.4577)
12,13-DiHOME	−5.9%	(0.1342)	−6.9%	(0.3002)	0.08	(0.5720)	−0.01	(0.9582)	−0.03	(0.8357)	0.10	(0.4579)
9,10,13-TriHOME	**6.3%**	**(0.0300)**	3.8%	(0.3761)	0.17	(0.1890)	−0.02	(0.8971)	**−0.29**	**(0.0296)**	0.22	(0.0709)
9,12,13-TriHOME	**6.1%**	**(0.0342)**	4.6%	(0.2777)	0.18	(0.1738)	−0.03	(0.8403)	0.11	(0.4990)	0.22	(0.0598)

^1^ Results are present as coefficients (Coef) and respective *p*-values. n = 45. ^2^ Analyzed using Poisson regression and presented as % change in count for each standard deviation change in the respective plasma-free oxylipins. ^3^ Analyzed using linear regressions and presented as standard deviation change in Y (pain frequency and intensity, functional aspects of chronic daily headache, and psychological aspects of chronic daily headache) for each standard deviation change in X (plasma-free oxylipins). ^4^ HIT-6, Headache Impact Test-6. Higher score = more headache impact on quality of life. ^5^ SF-12 (physical), Physical Health Composite Score of the Medical Outcomes Study Short Forms 12; SF-12 (mental), Mental Health Composite Score of SF-12. Higher score = better quality of life. ^6^ BSI-18, Brief Symptom Inventory. Higher score = more psychological distress. Data in bold: *p* < 0.05.

## Data Availability

The data presented in this study are available on request from the corresponding authors. The data are not publicly available due to restrictions that could compromise the privacy of research participants.

## Supplementary Materials

The following supporting information can be downloaded at: https://www.mdpi.com/article/10.3390/metabo13060690/s1, TableS1: List of oxylipins (total or free) analyzed in our prior and current dietary intervention studies [21,23,24,38]; Table S2: Parent ion, product ion, and retention time (RT) of 34 oxylipins analyzed by UPLC-MS/MS in this cohort [35].

## Abbreviations

AA	arachidonic acid
ALA	alpha-linolenic acid
BSI-18	Brief Symptom Inventory
CDHs	Chronic daily headaches
COX	cyclooxygenase
CYP	cytochrome
DGLA	di-homo-gamma-linolenic acid
DHA	docosahexaenoic acid
EDTA	ethylenediaminetetraacetic acid
EPA	eicosapentaneoic acid
HIT-6	Headache Impact Test-6
LA	linoleic acid
LOQ PUFA	limit of quantification Polyunsaturated fatty acid
sEH	soluble epoxide hydrolase
SF-12	Medical Outcomes Study Short Forms 12
SPE	solid phase extraction
TRPV	transient receptor potential vanilloid
UPLC-MS/MS	ultra high-pressure liquid chromatography-tandem mass spectrometry
