# Supplementary material for: Associations between Plasma Lipid Mediators and Chronic Daily Headache Outcomes in Patients Randomized to a Low Linoleic Acid Diet with or without Added Omega-3 Fatty Acids
Source: Metabolites. 2023 May 25;13(6):690. doi: 10.3390/metabo13060690 (PMC10304617; doi:10.3390/metabo13060690)
Supplement: Supplementary file 1 [file metabolites-13-00690-s001.zip › metabolites-2265942-supplementary.pdf]

# Supplementary information

**Supplementary Table S1.** List of oxylipins (total or free) analyzed in our prior and current dietary intervention. Oxylipins highlighted in yellow were not measured previously. They were measured in the present study. Asterisk (*) indicates that the oxylipin was measured but not detected.

| Oxylipins | Ramsden et al, 2013 [21] | Ramsden et al, 2017 [38] | Domenichiello et al, 2020 [23] | Ramsden et al, 2021 [24] | Current study |
|---|---|---|---|---|---|
| **Docosahexaenoic acid (DHA, 22:6 n-3)-derived oxylipins** | | | | | |
| 13(14)-EpDPE | / | / | / | / | free (plasma) |
| 16(17)-EpDPE | / | / | free (plasma) | / | free (plasma) |
| 19(20)-EpDPE | / | / | free (plasma) | / | free (plasma) |
| 10(11)-EpDPE | / | / | / | / | free (plasma)* |
| 7(8)-EpDPE | / | / | / | / | free (plasma)* |
| 7,8-DiHDPE | / | / | / | / | free (plasma)* |
| 10,11-DiHDPE | / | / | / | / | free (plasma) |
| 13,14-DiHDPE | / | / | / | / | free (plasma) |
| 16,17-DiHDPE | / | / | / | / | free (plasma) |
| 19,20-DiHDPE | / | / | / | / | free (plasma) |
| 4-HDHA | / | / | free (plasma) | total (plasma), free (serum) | / |
| 7-HDHA | / | / | free (plasma) | / | / |

| | | | | | |
|---|---|---|---|---|---|
| 10-HDHA | / | / | / | total (plasma), free (serum) | / |
| 14-HDHA | / | / | free (plasma) | total (plasma), free (serum) | / |
| 17-HDHA | free (plasma) | / | free (plasma) | total (plasma), free (serum) | / |
| RvD1 | / | / | free (plasma) | / | / |
| RvD2 | free (plasma) | / | / | / | / |
| **Eicosapentaneoic acid (EPA, 20:5 n-3)-derived oxylipins** | | | | | |
| 18-HEPE | free (plasma) | / | free (plasma) | / | / |
| 12-HEPE | / | / | / | / | free (plasma)* |
| 8(9)-EpETE | / | / | / | / | free (plasma)* |
| 11(12)-EpETE | / | / | / | / | free (plasma)* |
| 14(15)-EpETE | / | / | / | / | free (plasma)* |
| 17(18)-EpETE | / | / | / | / | free (plasma)* |
| 8,9-DiHETE | / | / | / | / | free (plasma)* |

| | | | | | |
|---|---|---|---|---|---|
| 11,12-DiHETE | / | / | / | / | free (plasma)* |
| 14,15-DiHETE | / | / | / | / | free (plasma)* |
| 17,18-DiHETE | / | / | / | / | free (plasma)* |
| Resolvin E1 | / | / | / | / | free (plasma)* |
| **α-linolenic acid (ALA, 18:3 n-3)-derived oxylipins** | | | | | |
| 9-HOTrE | / | / | free (plasma) | / | free (plasma) |
| 13- HOTrE | / | / | free (plasma) | / | / |
| 9(10)-EpODE | / | / | / | / | free (plasma) |
| 12(13)-EpODE | / | / | / | / | free (plasma) |
| 15(16)-EpODE | / | / | / | / | free (plasma) |
| 9,10-DiHODE | / | / | / | / | free (plasma) |
| 12,13-DiHODE | / | / | / | / | free (plasma)* |
| 15,16-DiHODE | / | / | / | / | free (plasma) |
| **Arachidonic acid (AA, 20:4 n-6)-derived oxylipins** | | | | | |
| 5-HETE | total (plasma) | / | free (plasma) | total (plasma), free (serum) | free (plasma) |
| 8-HETE | total (plasma) | / | free (plasma) | total (plasma), free (serum) | free (plasma) |

| | | | | | |
|---|---|---|---|---|---|
| 9-HETE | total (plasma) | / | / | / | free (plasma)* |
| 11-HETE | total (plasma) | / | free (plasma) | / | free (plasma) |
| 12-HETE | total (plasma) | / | free (plasma) | total (plasma), free (serum) | free (plasma) |
| 15-HETE | total (plasma) | / | free (plasma) | total (plasma), free (serum) | free (plasma) |
| 19-HETE | / | / | / | / | free (plasma)* |
| 20-HETE | / | / | free (plasma) | / | free (plasma)* |
| 5,6-DiHETrE | / | / | / | / | free (plasma)* |
| 8,9-DiHETrE | / | / | / | / | free (plasma) |
| 11,12-DiHETrE | / | / | / | / | free (plasma) |
| 14,15-DiHETrE | / | / | / | / | free (plasma)* |
| 5(6)-EpETrE | / | / | / | / | free (plasma)* |
| 8(9)-EpETrE | / | / | free (plasma) | / | free (plasma) |
| 11(12)-EpETrE | / | / | / | / | free (plasma) |
| 14(15)-EpETrE | / | / | free (plasma) | / | free (plasma) |

| | | | | | |
|---|---|---|---|---|---|
| 5-oxo-ETE | / | / | free (plasma) | / | free (plasma)* |
| 15-oxo-ETE | / | / | / | / | free (plasma)* |
| PGF2α | / | / | / | / | free (plasma) |
| PGE2 | / | / | / | free (serum) | free (plasma)* |
| PGD2 | / | / | / | / | free (plasma)* |
| PGB2 | / | / | / | / | free (plasma)* |
| PGJ2 | / | / | / | / | free (plasma)* |
| 15-deoxy-PGJ2 | / | / | / | / | free (plasma)* |
| TXB2 | / | / | / | free (serum) | free (plasma)* |
| LTB4 | / | / | / | free (serum) | free (plasma)* |
| LXA4 | / | / | / | / | free (plasma)* |
| 6-keto-PGF1a | / | / | / | / | free (plasma)* |

| | | | | | |
|---|---|---|---|---|---|
| Cysteinyl LTs | / | / | / | free (serum) | / |
| **Di-homo-gamma-linolenic acid (DGLA, 20:3 n-6)-derived oxylipins** | | | | | |
| 15(S)-HETrE | / | / | / | / | free (plasma) |
| **Linoleic acid (LA, 18:2 n-6)-derived oxylipins** | | | | | |
| 9-HODE | total (plasma) | / | free (plasma) | total (plasma), free (serum) | free (plasma) |
| 13-HODE | total (plasma) | / | free (plasma) | total (plasma), free (serum) | free (plasma) |
| 9-oxo-ODE | total (plasma) | / | / | / | free (plasma) |
| 13-oxo-ODE | total (plasma) | / | / | / | free (plasma)* |
| 9(10)-EpOME | / | / | free (plasma) | / | free (plasma) |
| 12(13)-EpOME | / | / | free (plasma) | total (plasma), free (serum) | free (plasma) |
| 9,10-DiHOME | / | / | free (plasma) | / | free (plasma) |
| 12,13-DiHOME | / | / | free (plasma) | total (plasma), free (serum) | free (plasma) |
| 9,10,13-TriHOME | / | / | / | total (plasma), free (serum) | free (plasma) |
| 9,12,13-TriHOME | / | / | / | total (plasma), free (serum) | free (plasma) |
| 9H-12,13E-LA | / | free (plasma) | / | total (plasma) | / |
| 9K-12,13E-LA | / | free (plasma) | / | / | / |

| | | | | | |
|---|---|---|---|---|---|
| 11H-12,13E-LA | / | free (plasma) | / | total (plasma) | / |
| 11H-9,10E-LA | / | free (plasma) | / | total (plasma), free (serum) | / |
| 13H-9,10E-LA | / | free (plasma) | / | total (plasma), free (serum) | / |

**Supplementary Table S2.** Parent ion, product ion, and retention time (RT) of 34 oxylipins detected by UPLC-MS/MS in this cohort. Optimization parameters derived from Hennebelle et al.[35]

| Oxylipins | Abbreviation | Parent ion m/z | Product ion m/z | RT |
|---|---|---|---|---|
| **Docosahexaenoic acid (DHA, 22:6 n-3)-derived oxylipins** | | | | |
| 13(14)-epoxydocosapentaenoic acid | 13(14)-EpDPE | 343.2 | 193.2 | 15.49 |
| 16(17)-epoxydocosapentaenoic acid | 16(17)-EpDPE | 343.2 | 233.2 | 15.37 |
| 19(20)-epoxydocosapentaenoic acid | 19(20)-EpDPE | 343.2 | 241.2 | 14.84 |
| 10,11-dihydroxydocosapentaenoic acid | 10,11-DiHDPE | 361.2 | 153.2 | 10.5 |
| 13,14-dihydroxydocosapentaenoic acid | 13,14-DiHDPE | 361.2 | 193.2 | 10.23 |
| 16,17-dihydroxydocosapentaenoic acid | 16,17-DiHDPE | 361.2 | 233.2 | 9.66 |
| 19,20-dihydroxydocosapentaenoic acid | 19,20-DiHDPE | 361.2 | 273.2 | 9.48 |
| **α-linolenic acid (ALA, 18:3 n-3)-derived oxylipins** | | | | |
| 9- hydroxyoctadecatrienoic acid | 9-HOTrE | 293.2 | 171.2 | 10.19 |
| 9(10)-epoxyoctadecadienoic acid | 9(10)-EpODE | 293.3 | 171.2 | 12.98 |
| 12(13)-epoxyoctadecadienoic acid | 12(13)-EpODE | 293.2 | 183.1 | 13.32 |
| 15(16)-epoxyoctadecadienoic acid | 15(16)-EpODE | 293.3 | 235.2 | 12.65 |

| | | | | |
|---|---|---|---|---|
| 9,10-dihydroxyoctadecadienoic acid | 9,10-DiHODE | 311.2 | 201.2 | 7.29 |
| 15,16-dihydroxyoctadecadienoic acid | 15,16-DiHODE | 311.2 | 223.2 | 7.22 |
| **Arachidonic acid (AA, 20:4 n-6)-derived oxylipins** | | | | |
| 5-hydroxyeicosatetraenoic acid | 5-HETE | 319.2 | 115.2 | 14.44 |
| 8-hydroxyeicosatetraenoic acid | 8-HETE | 319.2 | 155.2 | 13.57 |
| 11-hydroxyeicosatetraenoic acid | 11-HETE | 319.2 | 167.2 | 13.2 |
| 12-hydroxyeicosatetraenoic acid | 12-HETE | 319.2 | 179.2 | 13.57 |
| 15-hydroxyeicosatetraenoic acid | 15-HETE | 319.2 | 219.2 | 12.46 |
| 8,9-dihydroxyeicosatrienoic acid | 8,9-DiHETrE | 337.2 | 127.1 | 10.65 |
| 11,12-dihydroxyeicosatrienoic acid | 11,12-DiHETrE | 337.2 | 167.1 | 10.15 |
| 8(9)-epoxyeicosatrienoic acid | 8(9)-EpETrE | 319.2 | 155.2 | 15.98 |
| 11(12)-epoxyeicosatrienoic acid | 11(12)-EpETrE | 319.3 | 167.2 | 15.76 |
| 14(15)-epoxyeicosatrienoic acid | 14(15)-EpETrE | 319.2 | 219.3 | 15.1 |
| Prostaglandin F2-α | PGF2α | 353.2 | 309.2 | 4.54 |
| **Di-homo-gamma-linolenic acid (DGLA, 20:3 n-6)-derived oxylipins** | | | | |
| 15(S)-hydroxyeicosatrienoic acid | 15(S)-HETrE | 321.2 | 221.2 | 14.17 |
| **Linoleic acid (LA, 18:2 n-6)-derived oxylipins** | | | | |
| 9-hydroxyoctadecadienoic acid | 9-HODE | 295.2 | 171.1 | 12.3 |
| 13-hydroxyoctadecadienoic acid | 13-HODE | 295.2 | 195.2 | 12.22 |
| 9-oxo-octadecadienoic acid | 9-oxo-ODE | 293.2 | 185.1 | 13.38 |
| 9(10)-epoxyoctadecamonoenoic acid | 9(10)-EpOME | 295.3 | 171.1 | 15.16 |
| 12(13)-epoxyoctadecamonoenoic acid | 12(13)-EpOME | 295.3 | 195.2 | 14.9 |
| 9,10-dihydroxyoctadecamonoenoic acid | 9,10-DiHOME | 313.2 | 201.2 | 8.82 |
| 12,13-dihydroxyoctadecamonoenoic acid | 12,13-DiHOME | 313.2 | 183.2 | 8.45 |

| | | | | |
|---|---|---|---|---|
| 9,10,13-trihydroxyoctadecamonoenoic acid | 9,10,13-TriHOME | 329.2 | 171.1 | 4.57 |
| 9,12,13-trihydroxyoctadecamonoenoic acid | 9,12,13-TriHOME | 329.2 | 211.1 | 4.49 |

**References:**

[21] C.E. Ramsden, K.R. Faurot, D. Zamora, C.M. Suchindran, B.A. Macintosh, S. Gaylord, A. Ringel, J.R. Hibbeln, A.E. Feldstein, T.A. Mori, A. Barden, C. Lynch, R. Coble, E. Mas, O. Palsson, D.A. Barrow, J.D. Mann, Targeted alteration of dietary n-3 and n-6 fatty acids for the treatment of chronic headaches: a randomized trial, Pain, 154 (2013) 2441-2451.

[38] C.E. Ramsden, A.F. Domenichiello, Z.X. Yuan, M.R. Sapio, G.S. Keyes, S.K. Mishra, J.R. Gross, S. Majchrzak-Hong, D. Zamora, M.S. Horowitz, J.M. Davis, A.V. Sorokin, A. Dey, D.M. LaPaglia, J.J. Wheeler, M.R. Vasko, N.N. Mehta, A.J. Mannes, M.J. Iadarola, A systems approach for discovering linoleic acid derivatives that potentially mediate pain and itch, Science signaling, 10 (2017).

[23] A.F. Domenichiello, J.R. Jensen, D. Zamora, M. Horowitz, Z.-X. Yuan, K. Faurot, J.D. Mann, A.J. Mannes, C.E. Ramsden, Identifying oxidized lipid mediators as prognostic biomarkers of chronic posttraumatic headache, Pain, 161 (2020) 2775-2785.

[24] C.E. Ramsden, D. Zamora, K.R. Faurot, B. MacIntosh, M. Horowitz, G.S. Keyes, Z.-X. Yuan, V. Miller, C. Lynch, G. Honvoh, J. Park, R. Levy, A.F. Domenichiello, A. Johnston, S. Majchrzak-Hong, J.R. Hibbeln, D.A. Barrow, J. Loewke, J.M. Davis, A. Mannes, O.S. Palsson, C.M. Suchindran, S.A. Gaylord, J.D. Mann, Dietary alteration of n-3 and n-6 fatty acids for headache reduction in adults with migraine: randomized controlled trial, BMJ, 374 (2021) n1448.

[35] M. Hennebelle, Y. Otoki, J. Yang, B.D. Hammock, A.J. Levitt, A.Y. Taha, W. Swardfager, Altered soluble epoxide hydrolase-derived oxylipins in patients with seasonal major depression: An exploratory study, Psychiatry Res, 252 (2017) 94-101.
